# Access to New Clinic Appointments for Patients With Cancer

**DOI:** 10.1001/jamanetworkopen.2024.15587

**Published:** 2024-06-07

**Authors:** Debbie W. Chen, Mousumi Banerjee, Brittany Gay, Yi-Chun Wang, Lesley Miranda, Maya Watanabe, Christine M. Veenstra, Megan R. Haymart

**Affiliations:** 1 Division of Metabolism, Endocrinology, and Diabetes, University of Michigan, Ann Arbor; 2 Department of Biostatistics, University of Michigan, Ann Arbor; 3 School of Public Health, University of Michigan, Ann Arbor; 4 Center for Biostatistics in AIDS Research, Harvard T.H. Chan School of Public Health, Boston, Massachusetts; 5 Division of Hematology and Oncology, University of Michigan, Ann Arbor

## Abstract

**Importance:**

Racial and ethnic disparities have been observed in the outpatient visit rates for specialist care, including cancer care; however, little is known about patients’ experience at the critical step of attempting to access new clinic appointments for cancer care.

**Objective:**

To determine simulated English-speaking, Spanish-speaking, and Mandarin-speaking patient callers’ ability to access new clinic appointments for 3 cancer types (colon, lung, and thyroid cancer) that disproportionately impact Hispanic and Asian populations.

**Design, Setting, and Participants:**

This cross-sectional audit study was conducted between November 2021 and March 2023 using 479 clinic telephone numbers that were provided by the hospital general information personnel at 143 hospitals located across 12 US states. Using standardized scripts, trained research personnel assigned to the roles of English-speaking, Spanish-speaking, and Mandarin-speaking patients called the telephone number for a clinic that treats colon, lung, or thyroid cancer to inquire about a new clinic appointment. Data analysis was conducted from June to September 2023.

**Main Outcomes and Measures:**

The primary outcome was whether the simulated patient caller was able to access cancer care (binary variable, yes or no), which was defined to include being provided with a clinic appointment date or scheduling information. Multivariable logistic regression analysis was performed to determine factors independently associated with simulated patient callers being able to access cancer care.

**Results:**

Of 985 total calls (399 English calls; 302 Spanish calls; 284 Mandarin calls), simulated patient callers accessed cancer care in 409 calls (41.5%). Differences were observed based on language type, with simulated English-speaking patient callers significantly more likely to access cancer care compared with simulated Spanish-speaking and Mandarin-speaking patient callers (English, 245 calls [61.4%]; Spanish, 110 calls [36.4%]; Mandarin, 54 calls [19.0%]; *P* < .001). A substantial number of calls ended due to linguistic barriers (291 of 586 Spanish or Mandarin calls [49.7%]) and workflow barriers (239 of 985 calls [24.3%]). Compared with English-speaking simulated patient callers, the odds of accessing cancer care were lower for Spanish-speaking simulated patient callers (adjusted odds ratio [aOR], 0.34; 95% CI, 0.25-0.46) and Mandarin-speaking simulated patient callers (aOR, 0.13; 95% CI, 0.09-0.19). Compared with contacting clinics affiliated with teaching hospitals, callers had lower odds of accessing cancer care when contacting clinics that were affiliated with nonteaching hospitals (aOR, 0.53; 95% CI, 0.40-0.70).

**Conclusions and Relevance:**

In this cross-sectional audit study, simulated patient callers encountered substantial barriers when attempting to access clinic appointments for cancer care. These findings suggest that interventions focused on mitigating these barriers are necessary to increase access to cancer care for all patients.

## Key Points

**Question:**

What do patient callers experience when attempting to access a new clinic appointment for cancer care?

**Findings:**

This cross-sectional study of 479 clinic telephone numbers that were provided by the hospital general information personnel at 143 hospitals located across 12 states found that simulated patient callers were able to access cancer care in 409 of 985 total calls (41.5%). Multivariable logistic regression found that simulated patient callers had lower odds of accessing cancer care if they were Spanish- or Mandarin-speaking and if they were contacting a clinic that was affiliated with a nonteaching hospital.

**Meaning:**

These findings suggest that clinic appointment scheduling for new patients with cancer is a complex process that can inadvertently function as a gatekeeper to cancer care services for vulnerable patient populations, including patients with limited English proficiency; interventions focused on optimizing the clinic appointment scheduling workflow may improve access to cancer care for all patients.

## Teaser

This cross-sectional study analyzes linguistic and workflow barriers that prevent access to clinic appointments for patients with cancer.

## Introduction

In its 2001 report, *Crossing the Quality Chasm*, the Institute of Medicine outlined timely access to care as 1 of 6 domains that define quality health care.^1^ In the US, there is a well-established association of access to cancer care with improved health outcomes.^2,3,4^ Across the cancer care continuum, which spans from cancer prevention to the survivorship period,^5^ patients attempting to access cancer care need to successfully pass through multiple access points. These access points may include communication with the hospital general information personnel to obtain information about available cancer care services, communication with the clinic staff to schedule a new clinic appointment, transportation to and physical access into the clinic space and to ancillary services such as laboratories and imaging facilities, and communication with the health care team between appointments.^1,2,6^

Cancer disparities in the US are associated, in part, with differential access to cancer care services.^2,3,7,8,9,10^ However, the experiences of vulnerable patient populations, including patients with limited English proficiency (LEP), have been understudied. Prior work has identified linguistic barriers to accessing cancer care services at the level of the hospital general information personnel.^11^ In addition, prior work^12^ examining access to noncancer care services found a significant reduction in the ability of Spanish-speaking patient callers to schedule an outpatient appointment compared with their English-speaking counterparts. To effectively address disparities in access to cancer care services,^10^ it is necessary to identify barriers that patients encounter at the multiple access points across the cancer care continuum, including at the critical step of attempting to access a new clinic appointment for cancer care.

This audit study aimed to examine simulated patient callers’ access to a new clinic appointment for 3 cancer types (colon, lung, and thyroid cancer) that disproportionately impact Hispanic and Asian populations.^13,14^ In the US, Hispanic and Asian populations have the highest rates of LEP.^15,16^ While there is substantial diversity among Asian Americans, Chinese Americans make up the largest Asian ethnic group, and Mandarin is the most widely spoken Chinese dialect.^16,17,18^ In this context, our study included simulated patient callers who spoke English, Spanish, and Mandarin. We hypothesized that barriers related to the clinic’s workflow would prevent simulated patient callers from accessing cancer care, and that Spanish- and Mandarin- speaking simulated patient callers would be at risk for worse access due to the added burden of linguistic barriers that may arise.

## Methods

This cross-sectional audit study followed the Strengthening the Reporting of Observational Studies in Epidemiology (STROBE) reporting guideline. The University of Michigan institutional review board deemed this study exempt because it did not collect identifiable private information about individual members, employees, or staff of the organization.

Between November 15, 2021, and March 31, 2023, we conducted an audit study of 479 clinic telephone numbers that were provided by the hospital general information personnel at 143 hospitals located across 12 states that have varied proportions of residents with LEP (Arizona, California, Florida, Illinois, Massachusetts, Michigan, Missouri, New Jersey, New York, Oregon, Pennsylvania, and Texas). Simulated patient callers called the clinic telephone numbers during standard business hours (Monday through Friday between 8:00 AM and 5:00 PM local time) and inquired about a new clinic appointment for colon, lung, or thyroid cancer care. The clinic telephone number was provided by the hospital general information personnel in our prior study^11^ in which simulated English-speaking, Spanish-speaking, and Mandarin-speaking patient callers called the hospital general information telephone line to inquire about colon, lung, and thyroid cancer care services.

### Simulated Patient Call Protocol

Iterative pilot versions of the script with standardized responses, which were tested on clinics not included in the sample, informed the development of the final script and protocol. All simulated patient callers were female to reduce potential variation due to patient sex.

**Figure 1.**
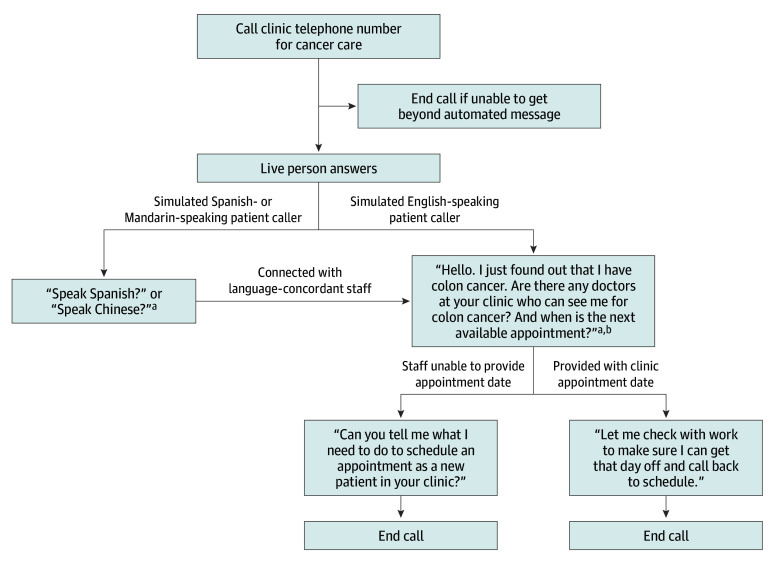
Standardized Script for Simulated Patient Callers In their respective assigned languages (English, Spanish, or Mandarin) and following the standardized script, simulated patient callers asked about a new clinic appointment for (1) colon cancer care, (2) lung cancer care, or (3) thyroid cancer care. ^a^The simulated patient callers ended the call if they were told “no” 3 times. ^b^For calls to clinics about lung and thyroid cancer care, the simulated patient callers replaced *colon* with *lung* and *thyroid*, respectively, in the script.

In their respective assigned languages (English, Spanish, or Mandarin) and following a standardized script, simulated patient callers asked about a new clinic appointment for (1) colon cancer, (2) lung cancer, or (3) thyroid cancer care (Figure 1). Although the standardized scripts were in each respective language, all simulated Spanish- and Mandarin-speaking patient callers started the telephone conversation using 2 English words, “Speak Spanish?” or “Speak Chinese?” respectively, to assist clinic staff and to simulate a more common clinical scenario. When assistance was provided by a language interpreter for communication with clinic staff, the Spanish- and Mandarin-speaking callers spoke in their respective languages with aid from the language interpreter.

Similar to a prior study that focused on evaluating access to cancer care at the level of the hospital general information telephone line,^11^ the script included standardized responses to common questions that could be asked. For example, if asked for the patient’s insurance, callers provided 1 of the 2 most common preferred provider organizations for each state as determined from data aggregated from state-specific and national-level sources.^19^ If asked questions that were not included in the script, the simulated patient callers provided standardized work-arounds (eg, if asked for insurance identifiers, callers stated that they did not have their insurance card with them during the call). All calls were kept as short as possible. All call information was entered in a REDCap online database upon completion of each call.

### Variables

The primary outcome was whether the simulated patient caller was able to access cancer care (binary variable, yes or no), which was defined to include the scenario in which the simulated patient caller was provided with (1) a clinic appointment date or (2) information on how to schedule a new clinic appointment. Duration of each call and the date of the clinic appointment, when provided by clinic staff, was recorded.

Similar to a prior study, states were categorized based on the size of each state’s population of residents with LEP per the 2010 US census (large population of residents with LEP, California, Florida, New York, and Texas; moderate population of residents with LEP, Arizona, Illinois, Massachusetts, and New Jersey; or small population of residents with LEP, Michigan, Missouri, Oregon, and Pennsylvania).^11,17^ Hospital and health system members of the Association of American Medical Colleges were classified as teaching hospitals.^20^

### Statistical Analysis

Logistic regression analysis and χ^2^ tests were used to test for univariate associations. A multivariable logistic regression model was used to determine which factors were independently associated with the primary outcome (simulated patient callers able to access cancer care). Analysis of variance was performed to compare the mean duration of the telephone calls, and the Mood median test was used to compare the median length of time from date of call to date of clinic appointment when provided by clinic staff. Statistical significance was defined as a 2-sided *P*  < .05. Statistical analyses were performed using R version 4.2.1 (R Project for Statistical Computing). Data analysis occurred from June to September 2023.

## Results

### Outcomes of Simulated Patient Calls

**Figure 2.**
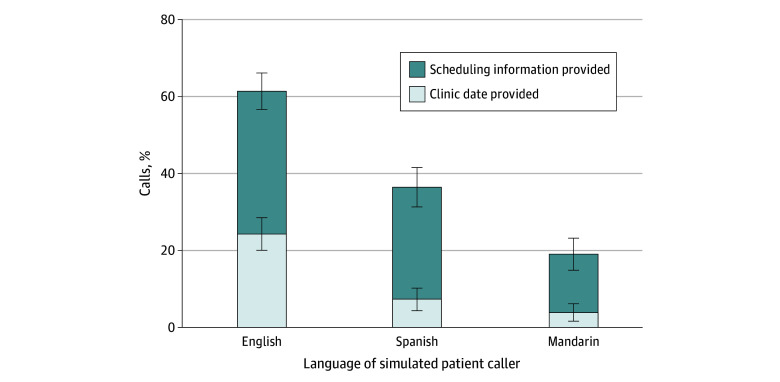
Simulated Patient Callers’ Access to Cancer Care by Language Type Simulated English-speaking patient callers were able to access cancer care in 245 of 399 calls (61.4%; clinic date provided, 97 of 399 calls [24.3%]; scheduling information provided, 148 of 399 calls [37.1%]). Simulated Spanish-speaking patient callers were able to access cancer care in 110 of 302 calls (36.4%; clinic date provided, 22 of 302 calls [7.3%]; scheduling information provided, 88 of 302 calls [29.1%]). Simulated Mandarin-speaking patient callers were able to access cancer care in 54 of 284 calls (19.0%; clinic date provided, 11 of 284 calls [3.9%]; scheduling information provided, 43 of 284 calls [15.1%]).

Of the 985 total calls (399 English calls; 302 Spanish calls; 284 Mandarin calls), simulated patient callers were able to access cancer care in 409 calls (41.5%). Differences were observed based on language type, with simulated English-speaking patient callers significantly more likely to access cancer care compared with their Spanish- and Mandarin-speaking counterparts (English, 245 calls [61.4%]; Spanish, 110 calls [36.4%]; Mandarin, 54 calls, [19.0%]; *P* < .001) (Figure 2). Among the 409 total calls in which simulated patient callers were able to access cancer care, the mean (SD) duration of the telephone call was greater for Spanish- and Mandarin-speaking callers (English, 5.98 [4.70] minutes; Spanish, 9.50 [6.95] minutes; Mandarin, 17.87 [10.30] minutes; *P* < .001).

Among the 130 total calls in which simulated patient callers were provided with a clinic appointment date, there was no significant difference in median time from date of call to date of clinic appointment by language type or by cancer care requested. Among the 279 total calls in which simulated patient callers were provided with information on how to schedule a new clinic appointment, the most common next steps were to submit a referral (165 calls [59.1%]), submit relevant medical records (97 calls [34.8%]), and/or to register as a new patient in the clinic’s electronic health record system (69 calls [24.7%]).

### Barriers to Accessing Cancer Care

**Figure 3.**
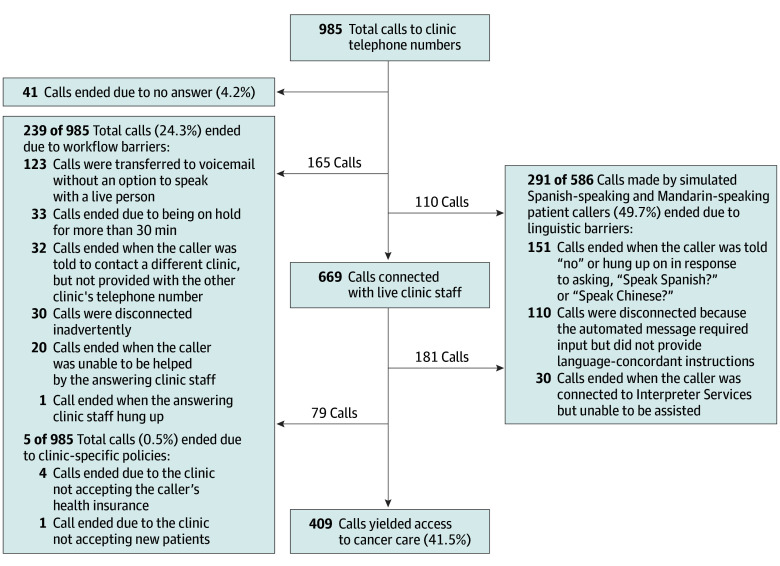
Barriers to Simulated Patient Callers Accessing a New Clinic Appointment for Cancer Care

Figure 3 summarizes the simulated patient callers’ experience of inquiring about a new clinic appointment for cancer care, with a focus on the barriers that prevented callers from accessing cancer care. Nearly one-quarter of the 985 total calls (239 calls [24.3%]) ended when simulated patient callers encountered workflow barriers. These barriers included (1) eventually going to a voicemail that did not provide the requested information (123 calls), (2) being on hold for more than 30 minutes (33 calls), (3) being told that a different clinic needed to be contacted but not provided with another telephone number to call (32 calls), (4) getting inadvertently disconnected (30 calls), and (5) unable to be helped by the answering staff member (21 calls).

Among the 586 calls made by the simulated Spanish- and Mandarin-speaking patient callers, nearly one-half (291 calls [49.7%]) ended due to linguistic barriers (Figure 3). The linguistic barriers included (1) the caller being told “no” or hung up on in response to asking, “Speak Spanish?” or “Speak Chinese?” (151 calls); (2) getting disconnected because the automated message required input but did not provide language-concordant instructions (110 calls); and (3) being unable to receive further assistance after getting connected to interpreter services (30 calls).

### Factors Associated With Access to Cancer Care

In univariate χ^2^ analyses, simulated patient callers were less likely to access cancer care if they were Spanish- or Mandarin-speaking (χ^2^_2_ = 127.44; *P* < .001) and if they were contacting a clinic that was affiliated with a nonteaching hospital (χ^2^_1_ = 10.99; *P* < .001) (Table). In multivariable logistic regression analysis, compared with English-speaking simulated patient callers, the odds of accessing cancer care were lower for Spanish-speaking simulated patient callers (adjusted odds ratio [aOR], 0.34; 95% CI, 0.25-0.46) and Mandarin-speaking simulated patient callers (aOR, 0.13; 95% CI, 0.09-0.19). Compared with contacting clinics affiliated with teaching hospitals, simulated patient callers also had lower odds of accessing cancer care when contacting clinics that were affiliated with nonteaching hospitals (aOR 0.53, 95% CI 0.40-0.70) (Table).

**Table.** Factors Associated With Simulated Patient Callers Successfully Accessing Cancer Care

Factor	Calls, No/Total No. (%) (N =985)		Univariate analysis, χ^2^ (df)	*P* value	Adjusted OR (95% CI)
	Able to access cancer care (n = 409)	Unable to access cancer care (n = 576)			
Language of caller					
English	245/399 (61.4)	154/399 (38.6)	127.44 (2)	<.001	1 [Reference]
Spanish	110/302 (36.4)	192/302 (63.6)			0.34 (0.25-0.46)
Mandarin	54/284 (19.0)	230/284 (81.0)			0.13 (0.09-0.19)
Size of the state’s population of residents with limited English proficiency^a^					
Large	134/329 (40.7)	195/329 (59.3)	0.16 (2)	.92	1 [Reference]
Moderate	128/308 (41.6)	180/308 (58.4)			0.98 (0.69-1.38)
Small	147/348 (42.2)	201/348 (57.8)			1.16 (0.83-1.63)
Hospital teaching status					
Teaching	207/436 (47.5)	229/436 (52.5)	10.99 (1)	<.001	1 [Reference]
Nonteaching	202/549 (36.8)	347/549 (63.2)			0.53 (0.40-0.70)
Cancer care requested					
Colon cancer	141/327 (43.1)	186/327 (56.9)	0.57 (2)	.75	1 [Reference]
Lung cancer	138/335 (41.2)	197/335 (58.8)			0.91 (0.65-1.28)
Thyroid cancer	130/323 (40.2)	193/323 (59.8)			0.86 (0.61-1.20)

Abbreviations: df, degrees of freedom; OR, odds ratio.

^a^ The size of each state's population of residents with limited English proficiency was based on the 2010 US census. States with a large population of residents with limited English proficiency included California, Florida, New York, and Texas; states with a moderate population of residents with limited English proficiency included Arizona, Illinois, Massachusetts, and New Jersey; and states with a small population of residents with limited English proficiency included Michigan, Missouri, Oregon, and Pennsylvania.

## Discussion

This cross-sectional study highlights existing barriers that patients may encounter when attempting to access a new clinic appointment for cancer care and illustrates how this access point in the cancer care continuum functions as a gatekeeper to cancer care services.^5^ In our study, simulated patient callers were able to access cancer care in fewer than one-half (41.5%) of the total calls. Furthermore, simulated Spanish-speaking and Mandarin-speaking patient callers were significantly less likely to access cancer care.

Differential access to cancer care services contributes to existing cancer disparities, with racially and ethnically minoritized patient populations and patients with LEP at risk for worse patient outcomes.^2,7,8,9,10,15^ Our current study, which focused on evaluating access to a new clinic appointment for cancer care, complements prior work that identified substantial linguistic barriers to cancer care access at the level of the hospital general information line,^11^ as well as a prior study identifying barriers to access in non-cancer settings.^12^ Thus, to improve access to cancer care for patients with LEP, our findings suggest that interventions to address linguistic barriers are needed at multiple access points along the cancer care continuum. In addition, we found that simulated patient callers had lower odds of accessing cancer care when calling clinic telephone numbers that were provided by the hospital general information personnel at nonteaching hospitals. While more research is needed to understand the reason for this observation, it may potentially be related to differential access to and availability of language-based resources. In a national survey of 861 hospitals,^21^ nonteaching hospitals were significantly less likely to report the availability of staff interpreters, external interpretation agencies, and language-based telephonic services than teaching hospitals.

### Strengths and Limitations

Strengths of our study include the use of simulated English-speaking, Spanish-speaking, and Mandarin-speaking patient callers; a focus on 3 cancer types that disproportionately impact Hispanic and Asian populations^13,14^; the inclusion of a heterogeneous cohort of clinics located in states with large, moderate, and small proportions of residents with LEP; and the inclusion of both teaching and nonteaching hospitals. In addition, the audit survey methodology, designed to reflect the patient’s experience of accessing cancer care, ensures generalizability of our results. Scheduling a new clinic appointment is a critical step to establishing care in any clinic.

Some potential limitations should be noted. First, we only assessed responses from the clinic telephone numbers that were provided by the hospital general information personnel. Patients seeking cancer care may also access clinic telephone numbers from other sources such as online websites. Second, the focus of this study was on evaluating access to cancer care only at the point of inquiring about a new clinic appointment for self-referred patients, and thus, may not reflect the experience of patients who are not self-referring or reflect the quality of cancer care once a patient is established in the clinic. Third, while we collected data on workflow barriers that were encountered by the simulated patient callers, our study did not capture all of the potential complexities of the clinic scheduling workflow. Fourth, our study findings may not be generalizable to all patients with cancer who speak a language other than English, including those who are able to obtain assistance from ad hoc language interpreters such as bilingual family members to facilitate the scheduling of a new clinic appointment.

## Conclusions

Our study provides actionable insight into existing linguistic and workflow barriers that patient callers may encounter when attempting to access a new clinic appointment for cancer care. Thus, there is a need for intervention to reduce these communication barriers and optimize the clinic appointment scheduling workflow. Otherwise, this access point in the cancer care continuum will continue to function as a gatekeeper to cancer care services, with many patient populations, including patients with LEP, unable to even get in the door to see a physician for their cancer care.

## Notes

Supplement.
Data Sharing Statement
