# Supplementary material for: Access to New Clinic Appointments for Patients With Cancer
Source: JAMA Netw Open. 2024 Jun 7;7(6):e2415587. doi: 10.1001/jamanetworkopen.2024.15587 (PMC11161839; doi:10.1001/jamanetworkopen.2024.15587)
Supplement: Supplement. — Data Sharing Statement [file jamanetwopen-e2415587-s001.pdf]

Data Sharing Statement

Chen. Access to New Clinic Appointments for Patients With Cancer. *JAMA Netw Open*. Published June 07, 2024. doi:10.1001/jamanetworkopen.2024.15587

Data
Data available: Yes
Data types: Deidentified participant data
How to access data: The deidentified data supporting this study will be available upon request after publication of this manuscript for research and ending 5 years following article publication. Requests should be directed to corresponding author (Debbie Chen, MD at chendeb@med.umich.edu). To gain access, data requestors will need to sign a data access agreement.
When available: With publication

Supporting Documents
Document types: None

Additional Information
Who can access the data: The deidentified data supporting this study will be available upon request after publication of this manuscript for research and ending 5 years following article publication. Requests should be directed to corresponding author (Debbie Chen, MD at chendeb@med.umich.edu). To gain access, data requestors will need to sign a data access agreement.
Types of analyses: The deidentified data supporting this study will be available upon request after publication of this manuscript for research and ending 5 years following article publication. Requests should be directed to corresponding author (Debbie Chen, MD at chendeb@med.umich.edu). To gain access, data requestors will need to sign a data access agreement.
Mechanisms of data availability: To gain access, data requestors will need to sign a data access agreement.
